# Exome sequencing in primary melanoma identifies novel drivers of melanoma progression

**DOI:** 10.1186/1479-5876-13-S1-P2

**Published:** 2015-01-15

**Authors:** Valentina Montagnani, Matteo Benelli, Alessandro Apollo, Silvia Pandolfi, Gianni Gerlini, Lorenzo Borgognoni, Barbara Stecca

**Affiliations:** 1 Laboratory of Tumor Cell Biology, Core Research Laboratory - Istituto Toscano Tumori, Florence, Italy; 2 Diagnostic Genetic Unit, Laboratory Department, Careggi University Hospital, Florence, Italy; 3 Plastic Surgery Unit, S.M. Annunziata Hospital - Regional Melanoma Referral Center, Istituto Toscano Tumori, Florence, Italy

## Background

Melanoma is the most aggressive skin cancer due to its high metastatic propensity and resistance to most traditional chemotherapeutic drugs [1,2]. At early stage melanoma can be cured by surgical excision, whereas metastatic melanoma is a highly lethal condition. To understand melanoma progression is crucial identify mutations that are involved in making an individual melanoma competent for metastatic spread. The most frequent known oncogenic mutation in melanoma is *BRAF*-V600E and several whole exome sequencing studies have revealed numerous other alterations [3-6]. It is well established that the aggressive behavior of melanoma is highly correlated with histological features, such as the thickness of the primary tumor and the mitotic index. Here we performed whole exome sequencing of 5 thin (<1mm in thickness) and 5 thick (>4mm in thickness) primary melanomas compared to matched-normal DNA.

## Materials and methods

We have collected 10 fresh primary melanomas from 10 untreated patients: DNA samples from melanoma tissues and peripheral blood (normal DNA) were available from all of the recruited patients. Genomic DNA was extracted from tumor and peripheral blood samples using the QIAamp DNA Minikit, (Qiagen, Hilden, Germany). Extracted DNA was used for Next-Generation Sequencing analysis by Illumina.

## Results

We confirmed recurrent somatic mutations in known melanoma-related genes, including BRAF, c-KIT, EGFR, PPP6C, MLL3 and several components of the glutamate signaling. In addition, we discovered mutations in genes not previously linked to this tumor, such as CSMD1, FGFR4 and components of the Hedgehog (HH) signaling pathway. In particular, in a thick melanoma we found a novel activating mutation in the transcription factor GLI1, one of the final effectors of the HH signaling. Additionally, we identified candidate metastasis-driving mutations such as ADAMTS6, ADAMTS7, CHD9, MLL3, NALCN and TSC2 in the 3 thick melanomas that produced metastasis. Interestingly, we identified several regions of focal somatic copy-number alterations (SCNAs) that were altered at significantly higher frequency in thick compared to thin melanomas. Several gene families are comprised among these regions of focal SCNAs, including components of Notch, HH and Wnt/β-catenin signaling pathways, BRAF, c-MYC and its cofactor PIM1, several ADAMs, EGFR and the HOX genes.

## Conclusion

Our data identify potential drivers of melanoma progression, enhancing our understanding of the genomic complexity underlying melanoma.
